# Lycopene Protects against Hypoxia/Reoxygenation Injury by Alleviating ER Stress Induced Apoptosis in Neonatal Mouse Cardiomyocytes

**DOI:** 10.1371/journal.pone.0136443

**Published:** 2015-08-20

**Authors:** Jiqian Xu, Houxiang Hu, Bin Chen, Rongchuan Yue, Zhou Zhou, Yin Liu, Shuang Zhang, Lei Xu, Huan Wang, Zhengping Yu

**Affiliations:** 1 Department of Cardiology, North Sichuan Medical College Affiliated Hospital, Nanchong 637000, Sichuan, China; 2 Department of Anesthesiology, North Sichuan Medical College Affiliated Hospital, Nanchong 637000, Sichuan, China; 3 Department of Occupational Health, Third Military Medical University, Chongqing 400038, China; 4 Department of physiology and pharmacology, Western University, London, Ontario, N6A 5C1, Canada; Virginia Commonwealth University, UNITED STATES

## Abstract

Endoplasmic reticulum (ER) stress induced apoptosis plays a pivotal role in myocardial ischemia/reperfusion (I/R)-injury. Inhibiting ER stress is a major therapeutic target/strategy in treating cardiovascular diseases. Our previous studies revealed that lycopene exhibits great pharmacological potential in protecting against the I/R-injury *in vitro* and *vivo*, but whether attenuation of ER stress (and) or ER stress-induced apoptosis contributes to the effects remains unclear. In the present study, using neonatal mouse cardiomyocytes to establish an *in vitro* model of hypoxia/reoxygenation (H/R) to mimic myocardium I/R *in vivo*, we aimed to explore the hypothesis that lycopene could alleviate the ER stress and ER stress-induced apoptosis in H/R-injury. We observed that lycopene alleviated the H/R injury as revealed by improving cell viability and reducing apoptosis, suppressed reactive oxygen species (ROS) generation and improved the phosphorylated AMPK expression, attenuated ER stress as evidenced by decreasing the expression of GRP78, ATF6 mRNA, sXbp-1 mRNA, eIF2α mRNA and eIF2α phosphorylation, alleviated ER stress-induced apoptosis as manifested by reducing CHOP/GADD153 expression, the ratio of Bax/Bcl-2, caspase-12 and caspase-3 activity in H/R-treated cardiomyocytes. Thapsigargin (TG) is a potent ER stress inducer and used to elicit ER stress of cardiomyocytes. Our results showed that lycopene was able to prevent TG-induced ER stress as reflected by attenuating the protein expression of GRP78 and CHOP/GADD153 compared to TG group, significantly improve TG-caused a loss of cell viability and decrease apoptosis in TG-treated cardiomyocytes. These results suggest that the protective effects of lycopene on H/R-injury are, at least in part, through alleviating ER stress and ER stress-induced apoptosis in neonatal mouse cardiomyocytes.

## Introduction

Myocardial ischemia/reperfusion (I/R)-injury remains a major clinical problem in these patients that receive therapies including percutaneous coronary intervention, thrombolysis, coronary artery bypass grafting and cardiac transplantation [1, 2]. Although conditions of myocardial reperfusion continue to improve with advances in perioperative cares and surgical techniques, there is still no effective therapy for preventing myocardial I/R-injury. Thus, the development of novel effective treatments for patients with cardiovascular diseases remains a major area of research. Myocardial I/R-injury is a complicated pathological process, its underlying mechanisms span a broad range of fundamental biological changes, including ionic, inflammatory, oxidant stress, mitochondria dysfunction and apoptosis. Unfortunately, these mechanisms have yet to be fully understood [3]. Recent studies have demonstrated that endoplasmic reticulum (ER) is closely associated with I/R-injury [4, 5].

ER is an extensive intracellular membranous network involved in protein synthesis, calcium homeostasis and highly sensitive to a wide variety of severe stress condition [6]. Stress condition such as a robust reactive oxygen species (ROS) generation can disrupt ER function, trigger ER stress and initiate the complex unfolded protein response (UPR) [7]. Once UPR is triggered, protein synthesis is blocked by activating eukaryotic translation initiation factor 2 subunit-α (eIF2α), IRE1α (inositol-requiring enzyme 1α) splices X-box binding proteins-1 (Xbp-1) mRNA and activation of activating transcription factor 6 (ATF6). Moreover, ER stress can be also elicited by the ER stress inducer thapsigargin (TG), which depletes Ca2+ from ER [8]. It has been reported that when ER stress is excessive and prolonged, it would increase transcription of pro-apoptotic C/EBP homology protein (CHOP) to mediate ER stress-induced apoptosis by regulating the expression of Bcl-2 family protein that located in the mitochondrial outer membrane [7, 9] and activate caspase-12, another pro-apoptotic factor, which involved in caspases cascade that is closely related to mitochondrial apoptotic pathway [5]. Thus, regulating ER stress and ER stress-induced apoptosis by targeting ROS generation may represent a promising therapeutic strategy for inhibiting I/R-injury.

Recently, much greater attention exploring potential drug candidates for ROS hypothesis-based therapy to prevent myocardial I/R-injury has been paid to natural and nontoxic antioxidants in clinical [9–11]. Lycopene, a member of the carotenoid family, is mostly found in tomatoes and other red fruits [12], has received much attention for being a highly efficient natural free radical scavenger and for its anti-tumor activity as well as neuroprotective effects [13–15]. Epidemiologic studies reported inverse associations between serum lycopene concentrations and risk of acute myocardial infarction (AMI) or sudden cardiac death [16, 17]. Indeed, lycopene has been shown to protect cardiomyocytes against myocardial I/R-injury through inhibiting oxidative stress and apoptosis [12, 18], our previous studies suggested that this cardioprotective effects may be attributed to its roles in improving the cellular ATP level and mitochondrial function [12, 19], but the exact mechanism has not been determined. It has been reported that AMP-activated protein kinase (AMPK), as a fuel sensor and modulator to modulate energy balance or oxidative stress at both cellular and whole body levels [20], will inactivate *in vivo* or *vitro* rat myocardial I/R model [21–24]. It has been well known that AMPK inactivation-mediated ER stress contributes to cardiomyocytes apoptosis and AMPK activation protects against I/R-injury via alleviating ER stress [21–24]. However, it is still unclear whether lycopene modulate ER stress and ER stress-induced apoptosis by activation of AMPK for alleviating myocardium I/R-injury.

Therefore, primary cultured mouse cardiomyocytes were used in this work to establish an *in vitro* model of hypoxia/reoxygenation (H/R) which simulates myocardium I/R *in vivo*, we hypothesized that alleviating ER stress or (and) ER stress-induced apoptosis through activating AMPK is a potential mechanism of lycopene in attenuating myocardial I/R injury. Our results add novel insights to this contentious field.

## Materials and Methods

### Ethics Statement

The animal care and procedures were strictly conformed to the National Institutes of Health Guide for the Care and Use of Laboratory Animals. This study was conducted with permission of Institutional Animal Care and Use Committee of the Third Military Medical University (Permit Number: SYXK (CQ) 2007–002).

### Antibodies and reagents

M199 medium and fetal bovine serum (FBS) were purchased from Hyclone (Logan, UT, USA). Dulbecco’s modified Eagle’s medium (DMEM) was purchased from Gibco (Carlsbad, CA, USA). Collagenases II and Annexin-V FITC/PI Apoptosis Detection kit were purchased from Invitrogen (San Diego, CA, USA). Streptomycin/penicillin, 2′, 7′-dichlorouoresce in diacetate (DCFH-DA), Hoechst 33342, RIPA lysis buffer, BCA Protein Assay kit, anti-mouse or anti-rabbit IgG antibody and rabbit anti-mouse Bip/GRP78 polyclonal antibody were supplied by Beyotime (Nanjing, China). Cell counting Kit-8 (CCK-8) was purchased from Dojindo Molecular Technologies (Kumamoto, Japan). Lycopene, Thapsigargin (TG), Dimethylsulphoxide (DMSO) and β-actin antibody were purchased from Sigma-Aldrich (St. Louis, MO, USA). In situ cell death detection kit was purchased from Roche Diagnostics Corp (IN, USA). Rabbit anti-Bax, rabbit anti-Bcl-2, mouse anti-caspase-3, rabbit anti-phospho AMPKα, rabbit anti-AMPKα antibodies, rabbit anti-phospho-eIF2α and rabbit anti-eIF2α antibodies were obtained from Cell Signaling Technology (Danvers, MA, USA). Rabbit-ATF6α polyclonal antibody was purchased from Santa Cruz Biotechnology Inc. (Santa Cruz, CA, USA). Super Signal West Pico blotting was purchased from Pierce (Rockford, USA). Rabbit anti-mouse CHOP/GADD153 polyclonal antibody was purchased from Abcam (Cambridge, MA, USA). PrimeScript RT master mix and PrimeScript RT reagent kit with gDNA eraser were purchased from TaKaRa Biotechnology (Otsu Shiga, Japan). Rabbit anti-mouse caspase-12 polyclonal anti-body was obtained from Biovision (CA, USA).

### Cell culture and treatment

Primary neonatal cardiomyocytes from 1-day-old Neonatal C57BL/6 mouse (Experimental Animal Center of the Third Military Medical University, Chongqing, China) were isolated by enzymatic disassociation as previously described with some modifications [12, 25, 26]. Briefly, the hearts were cut into small pieces and washed, followed by digestion with 0.1% collagenases type II for 15 min in a 5% CO2 humidified atmosphere at 37°C, these cells were resuspended in M199 medium containing 10% fetal bovine serum and 1% streptomycin/penicillin. These steps were repeated until the tissue fragments disappeared. Isolated cells were first filtered with a 70μm cell strainer (Millipore, Billerica, MA, USA) and then centrifuged 300g for 5 min at room temperature to concentrate them in cells pellet, the cells were resuspended in the M199 medium and preplated on uncoated well plates at 37°C for 1h to enrich the culture with cardiomyocytes. The non-adherent cardiomyocytes were collected and plated on 1% gelatin-coated well plates, and then cultured for 48–72h. Cardiomyocytes were hypoxia for 4h, followed by different times (1, 2, 4, 6, 8 and 12h) for reoxygenation to establish a H/R model, and then divided into control, lycopene, H/R and lycopene+H/R group as described in our previous publication [12]. To further investigate the effects of lycopene on ER stress-induced injury, TG was used to establish a ER stress model and cardiomyocytes were pretreated with TG (0.25, 0.5, 0.75 and 1.0μM) for 10h to confirm a suitable dose, in addition, cardiomyocytes were treatment with 0.5μM TG for 2, 4, 8, 10 and 20h to confirm a specific time, after that cardiomyocytes were divided into control, TG, lycopene and lycopene+TG group. TG was dissolved in DMSO and applied after the lycopene pretreatment in TG and lycopene+TG group, the other groups received an amount of DMSO equal to that present in TG-treated ones.

### Cell Viability Assay

Cell viability was assessed using the CCK-8 according to the manufacturer’s instructions. In brief, 3×105 cells per well were seeded into 96-well plates in 4 replicates. After the indicated treatments, 10μl CCK-8 solution was added to each well, and then the cells were incubated at 37°C for 4h in the incubator, after that the optical density(OD) at 450 nm was measured using an Infinite 200 Microplate Reader (Tecan, M200). The experiment was repeated three times. Data were given as percentages of control group.

### Assessment of Apoptosis

Early apoptosis and late apoptosis/necrosis were detected using an Annexin-V FITC/PI Apoptosis Detection kit according to the manufacturer’s instruction. Briefly, cardiomyocytes were seeded into a 12-plate at a density of 5×105/well, after the indicated treatment, adherent cells were enzymatically digested for 50S with 0.25% trypsin and collected together with floating dead cells. Approximately 1×106 cells were washed twice with cold PBS, and then resuspended in 200μl cold 1×binding buffer containing 5μl of Annexin V-FITC and 5μl of PI. Cells were incubated at room temperature for 15 min in the dark and fluorescence was analyzed using a FACSCalibur flow cytometer (Becton Dickinson, San Jose, CA, USA). At least 10,000 events were recorded. Apoptotic cells were expressed as a percentage of the total number of cells. Apoptosis of cardiomyocytes were analyzed with an in situ cell death detection kit as described in our previous publication [12]. The staining was observed under a Zeiss confocal laser scanning microscope (Carl Zeiss, LSM 780 Meta Confocal Laser Scanning Microscope). The experiment was repeated three times.

### Measurement of Intracellular ROS generation

Intracellular ROS production was determined using flow cytometry and confocal laser scanning microscope respectively. For flow cytometry analyses [27], 5×105 cells per well were seeded into the 12-plate, after the indicated treatment, the cells were incubated with freshly prepared DCFH-DA at 37°C for 30 min in the dark, and then cells were harvested as the above described. Finally, cells were analyzed immediately by flow cytometry at 488 nm for excitation and 530 nm for emission. The data were analyzed using the Cell Quest software (Becton Dickinson, San Jose, CA, USA). For confocal laser scanning microscope determination [28], 3×105 cells/well were seeded into the glass-bottomed dishes. After the indicated treatment, the ROS was labeled with DCFH-DA probe at 37°C for 30 min in the dark. Then the cells were rinsed twice and Hoechst 33342 was added to see nuclear staining. Finally, Cells were rinsed three times with PBS to remove Hoechst 33342 and imaged using a Zeiss confocal laser scanning microscope (Carl Zeiss, LSM 780 Meta Confocal Laser Scanning Microscope). The mean fluorescence intensity (MFI) of the ROS-positive (green) cells from four different fields was averaged using LSM Image Examiner software for statistical analysis. These experiments were repeated for three times, and cellular fluorescence intensity was expressed as the fold change to the control group.

### Quantitative Real-time PCR

Total RNA was isolated from cultured cardiomyocytes using the Trizol reagent [29] (TaKaRa, Japan). RNA samples (1μg) were reverse transcribed to cDNA using PrimeScript RT reagent kit with gDNA eraser according to the manufacturer’s instruction. The mRNA level was determined with the Bio-Rad CFX96 Real-time PCR system-C1000 Thermal Cycler [29] (Bio-Rad Laboratories, Hercules, CA, USA), The thermal cycle conditions from SYBR Premix Ex Taq II assay (TaKaRa, Japan) were as follows: 30s at 95°C, followed by 40 cycles of 95°C for 30s, 60°C for 30s and 72°C for 40s. The primers sequences for GRP78, ATF6, eIF2α, uXbp-1, sXbp-1, GADD153, caspase-12 and GAPDH were designed as described previously [30–33]. The mRNA levels were quantitated first against GAPDH levels in the same sample, then normalized to control group which was set up as 1-fold. These experiments were repeated for three times.

### Western blotting analysis

Protein extraction and western blotting analysis was performed as previously described [34, 35]. 60μg protein lysate was separated by 12% SDS-PAGE and then transferred to polyvinylidene difluoride (PVDF) membranes (Millipore. Billerica, MA). Non-specific binding were blocked for 1.5h at 37°C in TBST with 5% fat-free milk. The membranes were incubated with primary antibodies against Bip/GRP78 (1:1,000), CHOP/GADD153 (1:500), Bax (1:1,000), Bcl-2 (1:1,000), caspase-12 (1:500), caspase-3 (1:1,000), AMPK (1:1,000), p-AMPK (1:1,000), eIF2α (1:1,000), p-eIF2α (1:1,000), ATF6α (1:1,000) and β-actin antibody (1:5,000) at 4°C overnight and then with an anti-mouse or anti-rabbit IgG antibody (1:1,000) for 1.5h. The membrane was visualized by enhanced chemiluminescence using Super Signal West Pico blotting detection reagents. Each experiment was repeated at least three times.

### Statistical analysis

Data are expressed as mean ± SEM. Statistical analyses were performed with GraphPad Prism 5.0 software (GraphPad Software, Inc., SanDiego, CA). Data were analyzed with one-way ANOVA followed by post hoc least significant difference (LSD) tests. Multiple group comparisons were determined by using Newman-Keuls Multiple Comparison Test. *P*<0.05 was accepted as statistically significant.

## Results

### Lycopene alleviated H/R-injury

**Fig 1:**
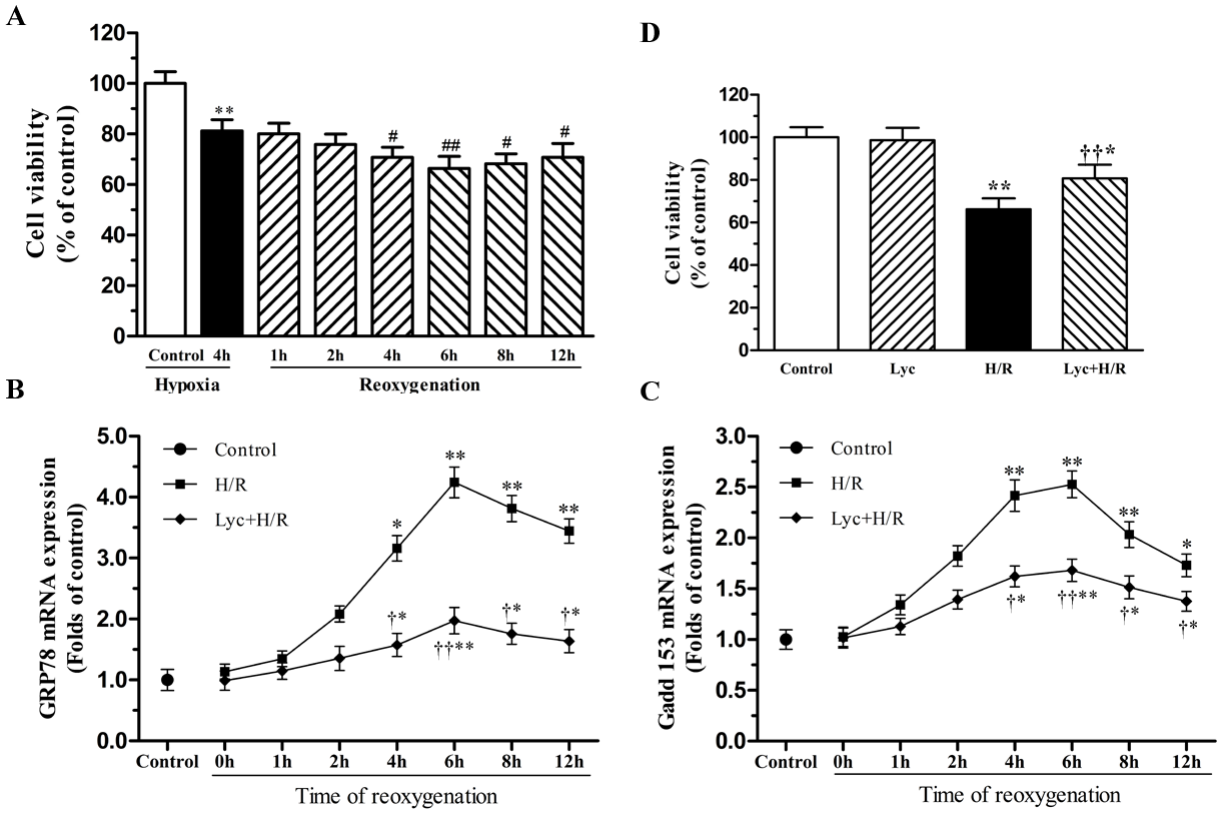
Effects of lycopene on H/R-induced loss of cell viability and ER stress. Primary cultured neonatal mouse cardiomyocytes were pre-incubation with or without 5μM lycopene for 4h prior to 4h of exposure to hypoxia condition with serum-free and glucose-free medium, followed by 1, 2, 4, 6, 8 or 12h of reoxygenation in normal medium. Cell viability was determined by CCK-8 assay. The total mRNA of cardiomyocytes was extracted, and the mRNA levels of GRP78 and GADD153 were detected by Real-time PCR. (A) Effects of H/R on cell viability. (B) Time-dependent effect of lycopene on GRP78 mRNA expression in H/R-treated cardiomyocytes. (C) Time-dependent effect of lycopene on GADD153 mRNA expression in H/R-treated cardiomyocytes. (D) Lycopene reversed H/R-evoked loss of cell viability. Cells were pretreated with or without 5μM lycopene for 4h prior to H/R treatment. Results were presented as a percentage of the control cell survival (set to 100%). Values are the mean ± SEM from three independent experiments. *P<0.05,**P<0.01 versus control; ^***#***^ *P*<0.05, ^***##***^ *P* <0.01 versus hypoxia, ^**†**^ *P*<0.05, ^**††**^ *P*<0.01 versus H/R group. (Lyc, Lycopene. H/R, hypoxia/reoxygenation).

Cultured cardiomyocytes underwent 4h of hypoxia followed by 1, 2, 4, 6, 8 or 12h of reoxgenation, cell viability was then assessed by CCK-8. As shown in Fig 1A, cell viability further decreased after reoxgenation compared with the 4h hypoxia group, among that treatment with 4h hypoxia and 6h reoxgenation significantly reduced cell viability to 66.30±4.84% of control. To efficiently conduct the following experiment, we determined whether the ER stress is activated in response to H/R. This concentration was determined based on our previous study [12], and then underwent the above H/R treatment, the expression of GRP78 and CHOP/GADD153 mRNA, representative molecular of ER stress, were detected by Real-time PCR. As illustrated in Fig 1B and 1C, the GRP78 and CHOP/GADD153 mRNA expression had significant increase in the 4th hour of reoxygenation and had peaked in the 6th hour of reoxygenation, whereas lycopene could alleviate these changes. Therefore, we selected 4H/6R for the following experiments.

**Fig 2:**
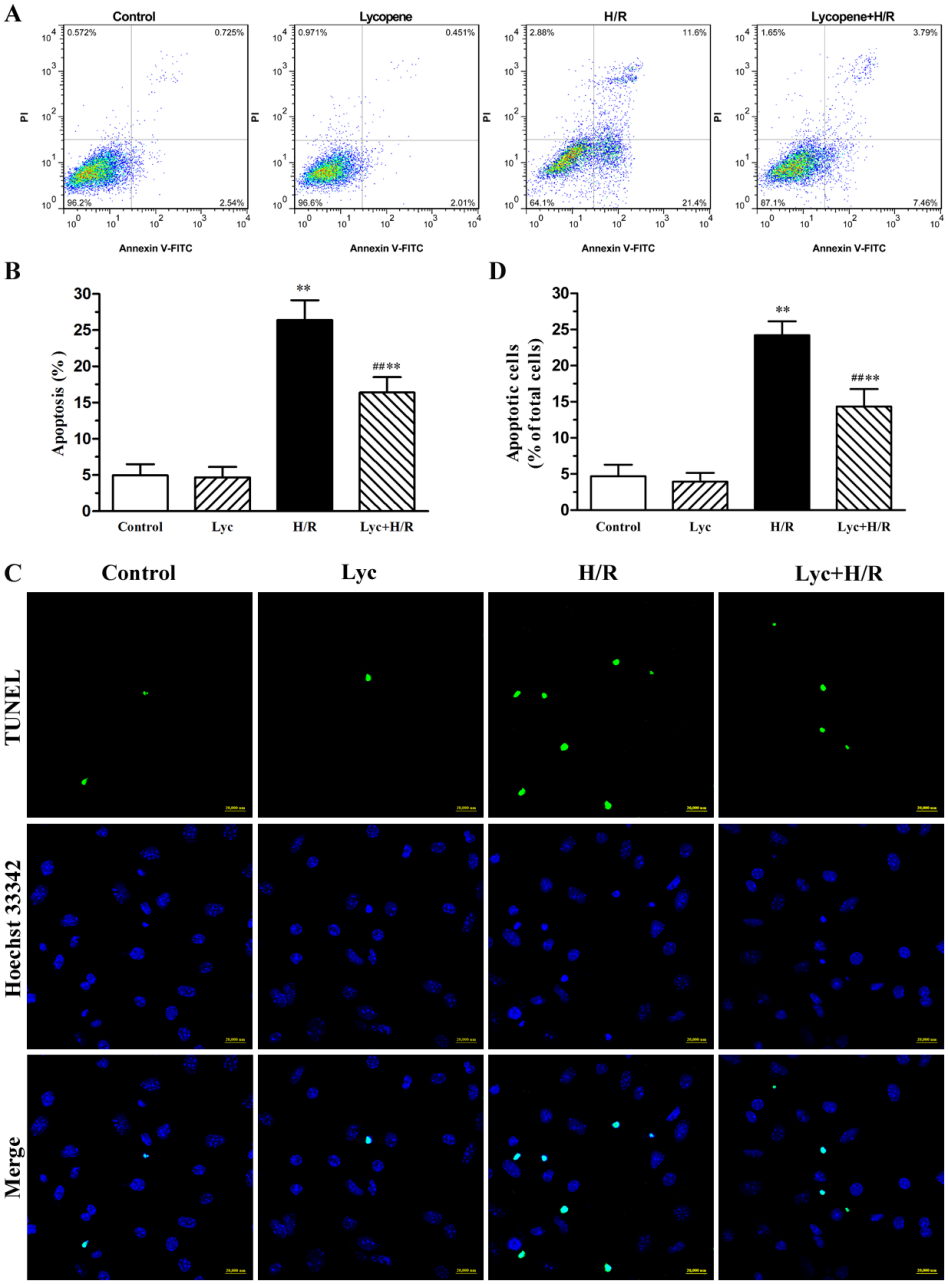
Effects of lycopene on H/R-induced apoptosis in cardiomyocytes. Cardiomyocytes were pretreated with or without 5μM lycopene for 4h prior to H/R treatment. (A) Apoptotic cells were detected with flow cytometry by AnnexinV-FITC and PI counterstaining, the representative flow cytometry figures are from each group. (B) The statistical graph of apoptosis rate with AnnexinV-FITC and PI counterstaining are from three independent experiments. (C) Morphology characteristic of cardiomyocytes apoptosis was determined by staining with TUNEL and Hoechst 33342, representative images of TUNEL-positive cells (green, top row) and Hoechst counterstaining cells (blue, middle row) are shown in these representative fields. Scale bar: 20μm. (D) Quantitative analysis of TUNEL staining was from three independent experiments. The histogram shows the relative proportion of TUNEL-positive cells in the different groups. Values are mean±SEM. *P<0.05, **P<0.01 versus control; ^**##**^ *P* <0.01 versus H/R group. (Lyc, Lycopene. H/R, hypoxia/reoxygenation).

As shown in Fig 1D, lycopene has no significant toxic effects on the cell viability of cultured cardiomyocytes when applied alone. A significant amelioration to the loss of cell viability is observed in this process of H/R when lycopene pretreated cells. The loss of cell viability is routinely accompanied by apoptosis. Here, we found that lycopene significantly decreased the percentage of apoptotic cells in H/R-treated cardiomyocytes by using Annexin-V-FITC and PI staining (Fig 2A and 2B). Conversely, lycopene alone did not show any effects on apoptosis. The protective effect of lycopene against apoptosis was further confirmed by TUNEL staining, typical examples are shown in Fig 2C and 2D, exposure to H/R caused a significant increase in the numbers of apoptotic cardiomyocytes relative to control and lycopene group. Nonetheless, pre-incubation with lycopene markedly diminished H/R-induced cell apoptosis.

### Lycopene suppresses ROS generation in H/R-treated cardiomyocytes

**Fig 3:**
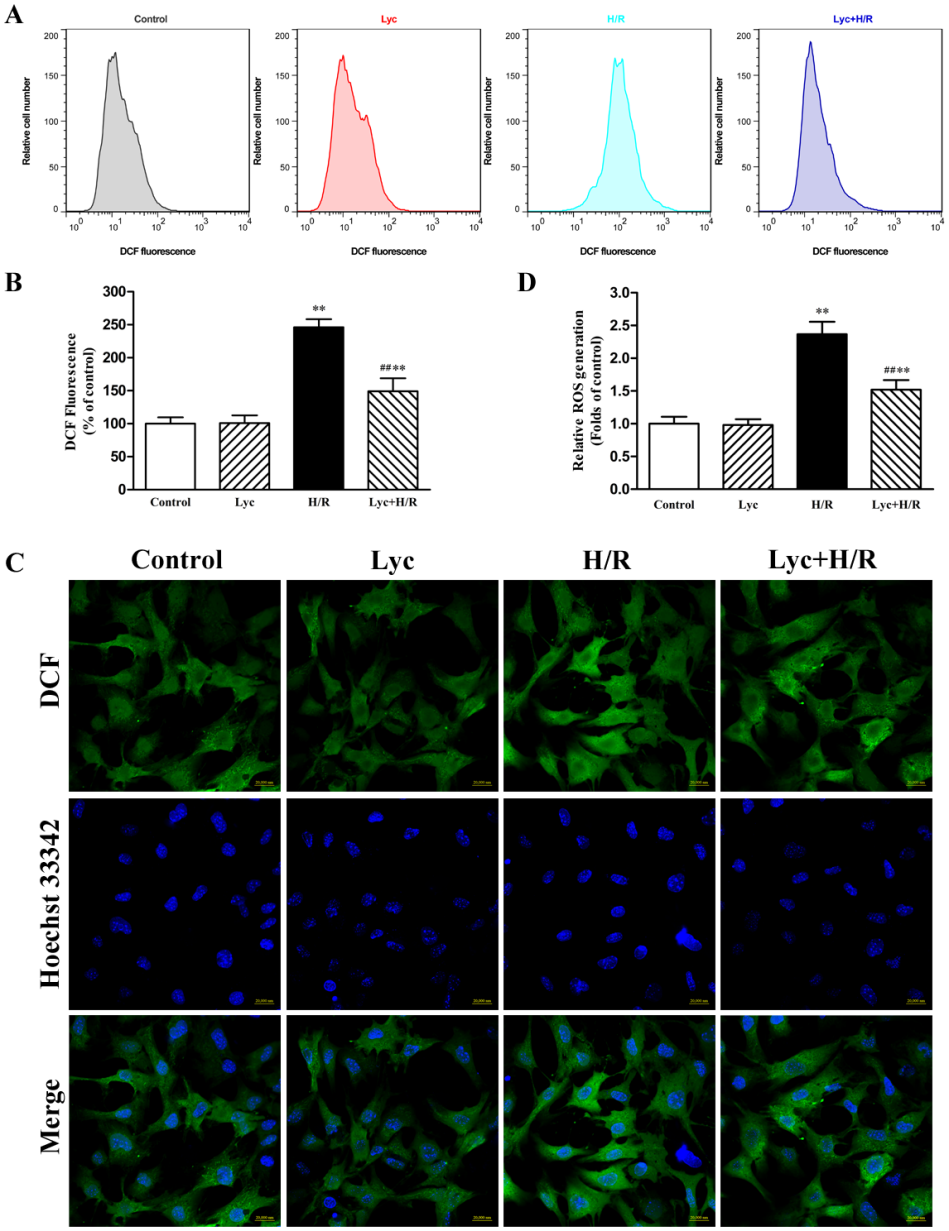
Effects of lycopene on ROS generation in cardiomyocytes. Neonatal cardiomyocytes were treated with or without 5μM lycopene for 4h prior to H/R treatment. ROS levels were visualized by DCF staining. (A) ROS generation was evaluated with flow cytometry using DCF fluorescence, the representative flow cytometry figures are from different groups. (B) The fluorescence intensity of the cells was calculated relative to that of control cells using flow cytometry from three independent experiments. (C) ROS production labeled by DCF fluorescence was monitored with confocal microscopy, representative images of ROS-positive cells (green, top row) and Hoechst counterstaining cells (blue, middle row) are shown in their representative fields. Scale bar: 20μm. (D) Quantitative analysis of the mean fluorescence intensity (MFI) from four different confocal microscopy scope fields in each group were averaged, the assays were repeated three times. Values are presented as mean ± SEM. **P<0.01 versus control, ^**##**^ *P* <0.01 versus H/R group. (Lyc, Lycopene. H/R, hypoxia/reoxygenation).

It has been well known that the protective effect of lycopene against apoptosis is to quench ROS in various cell types [12, 36]. Accordingly, we measured intracellular ROS using DCFH-DA and flow cytometry. As illustrated in Fig 3A and 3B, the cardiomyocytes produced intense fluorescent signals in H/R-treated cultures, indicating a significant increase in ROS production. On the contrary, pretreatment with lycopene significantly decreased H/R-induced ROS generation. The data above were also confirmed by confocal microscopy, typical figures are shown in Fig 3C and 3D, H/R-treated cardiomyocytes showed much more bright green fluorescence than the control and lycopene group. In contrast, pre-incubation with lycopene showed weakened green fluorescence intensity in H/R-treated cardiomyocytes.

### Lycopene attenuates ER stress through activating AMPK in H/R-treated cardiomyocytes

**Fig 4:**
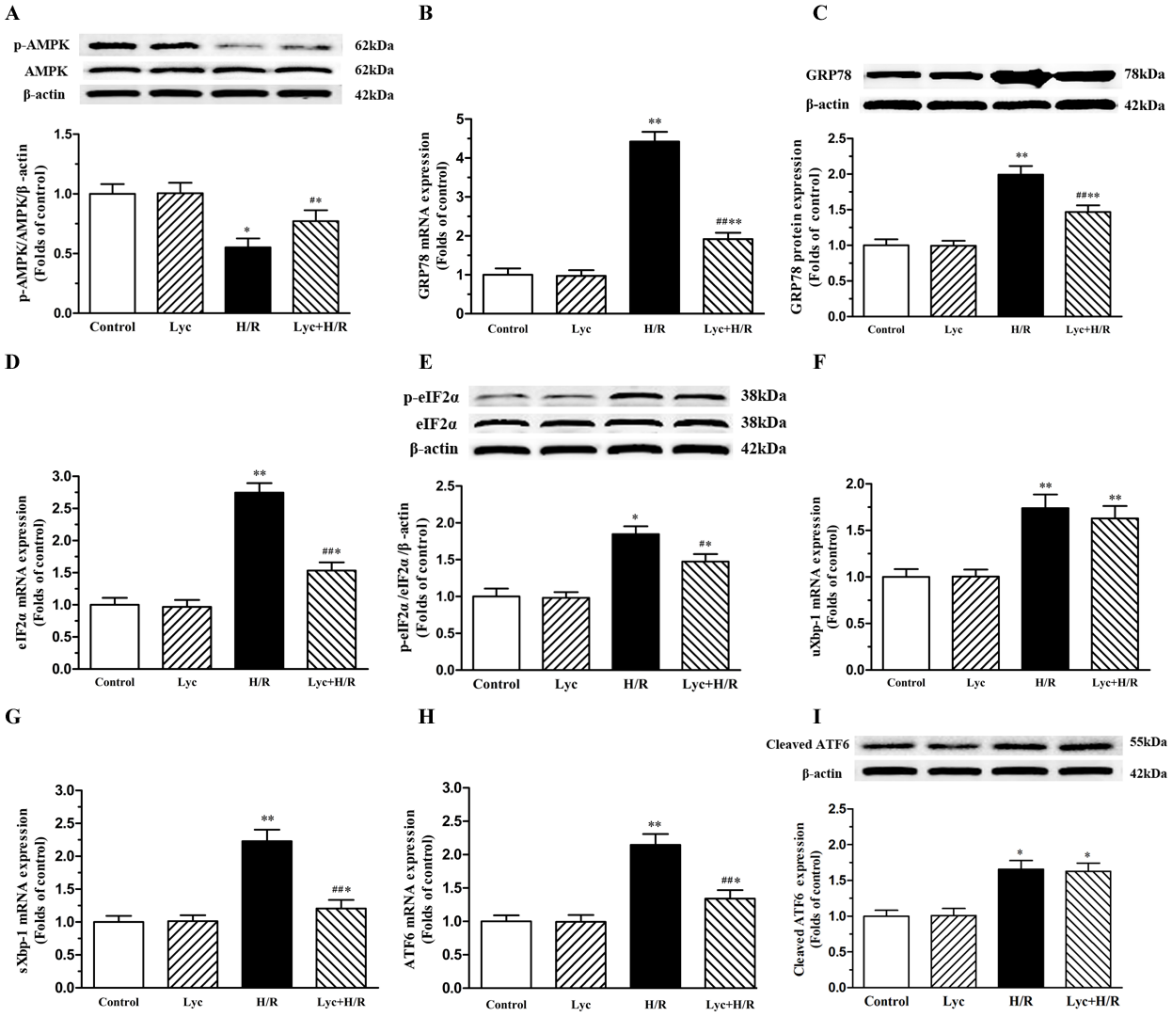
Effects of lycopene on AMPK phosphorylation and ER stress in cardiomyocytes. Cardiomyocytes were pretreated with or without 5μM lycopene for 4h prior to H/R treatment. The total mRNA of cardiomyocytes in each group was extracted and the mRNA levels of GRP78, eIF2α, ATF6, uXbp-1 and sXbp-1 were determined by Real-time PCR. The total proteins were used to determine the protein levels of Bip/GRP78 and cleaved ATF6, the phosphorylated levels of AMPK and eIF2α, representative western blot images and quantification analysis normalized to the β-actin and control, respectively, are from three independent experiments. (A) Effects of lycopene on AMPK phosphorylation in cardiomyocytes. (B) Effects of lycopene on H/R-induced GRP78 mRNA expression. (C) Effects of lycopene on Bip/GRP78 protein in H/R-treated cardiomyocytes. (D) Effects of lycopene on H/R-induced eIF2α mRNA expression. (E) Effects of lycopene on eIF2α phosphorylation in cardiomyocytes. (F) Effects of lycopene on the unspliced Xbp-1 mRNA expression in cardiomyocytes. (G) Effects of lycopene on the spliced Xbp-1 mRNA expression in cardiomyocytes. (H) Effects of lycopene on H/R-induced ATF6 mRNA expression. (I) Effects of lycopene on the cleaved ATF6 in cardiomyocytes. Results are expressed as the mean ± SEM. *P<0.05, **P<0.01 versus control; ^***#***^ *P*<0.05, ^**##**^ *P* <0.01 versus H/R group. (Lyc, Lycopene; H/R, hypoxia/ reoxygenation).

Previous studies have established that ROS may be mediate injury through inactivating AMPK [20, 21]. To investigate whether lycopene can modulate the activity of AMPK in H/R-treated cardiomyocytes, we therefore evaluated the phosphorylation of AMPK using western blot. As shown in Fig 4A, H/R treatment significantly decreased the phosphorylation of AMPK and lycopene pretreatment up-regulated the phosphorylated AMPK. It has been reported that AMPK inactivation could mediate ER stress in myocardial I/R injury [21–24], the maker of ER stress was therefore detected. We noticed that the levels of GRP78 mRNA and proteins were markedly increased in H/R-treated cardiomyocytes compared with the control group. Pretreatment of cells with lycopene significantly abrogated H/R-induced robust increase in GRP78 mRNA and protein levels (Fig 4B and 4C). Meanwhile the expression of the three ER stress arms was evaluated. eIF2α, a downstream target of the PERK pathway, the mRNA expression and the level of phosphorylation were increased in H/R-treated cardiomyocytes compared to the control group, whereas lycopene ameliorated these changes (Fig 4D and 4E). Xbp-1, a downstream target of the IRE1 pathway, the unspliced and spliced forms of Xbp-1 mRNA were measured using Real-time PCR, the level of the unspliced and spliced Xbp-1 mRNA in cardiomyocytes were increased in H/R-treated cells and lycopene significantly reversed the increase of the spliced Xbp-1 mRNA, but not the unspliced form of Xbp-1 (Fig 4F and 4G). Furthermore, the mRNA expression of ATF6 and the cleaved ATF6 had significant increase in H/R-treated cells, lycopene significantly decreased the increase trend of ATF6 mRNA, but not the cleaved ATF6 (Fig 4H and 4I).

### Lycopene ameliorates ER stress-induced apoptosis in H/R-treated cardiomyocytes

**Fig 5:**
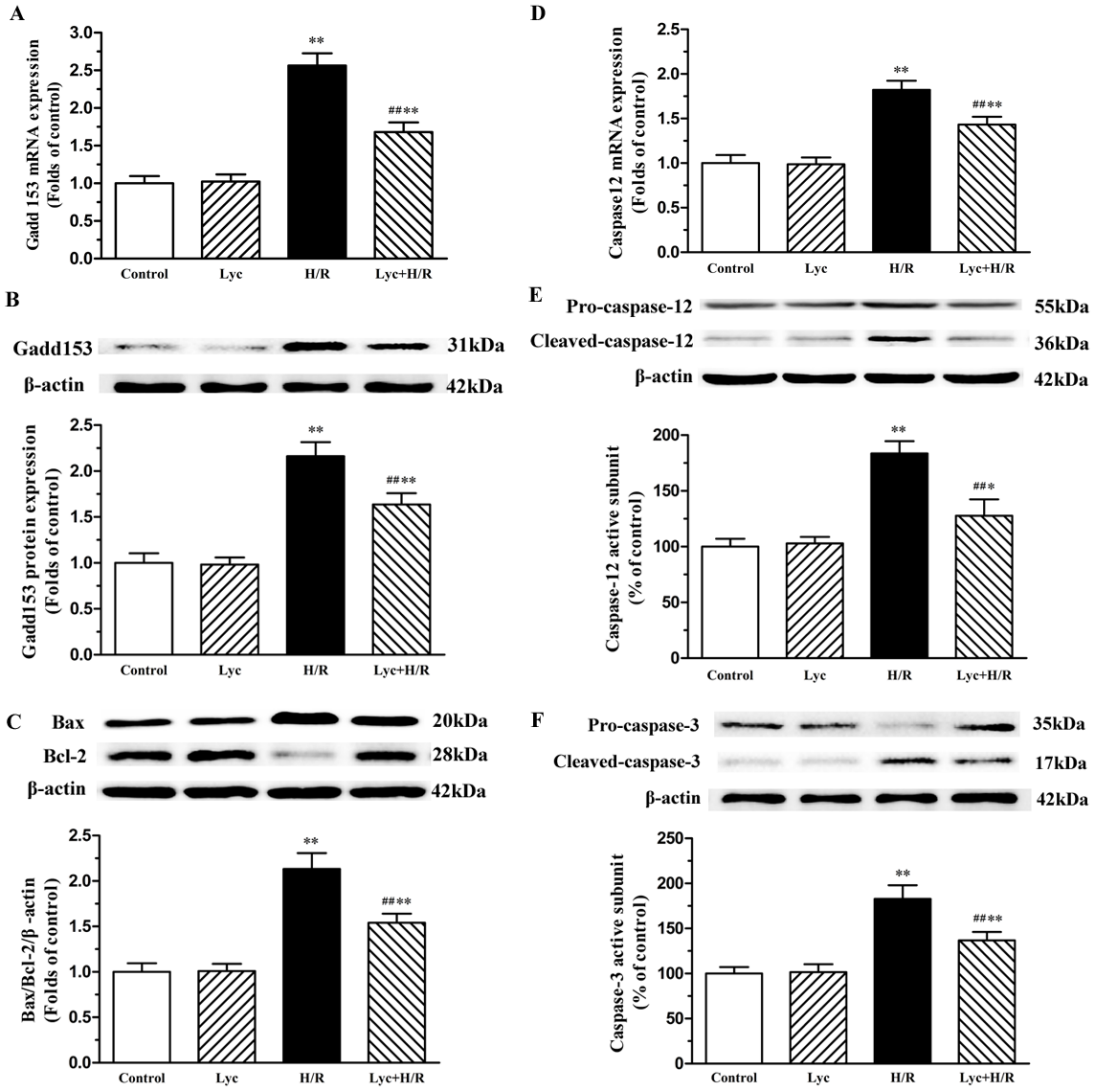
Effects of lycopene on ER stress apoptotic molecules in H/R-treated cardiomyocytes. Cardiomyocytes were pretreated with or without 5μM lycopene for 4h prior to H/R treatment. The total mRNA of cardiomyocytes was extracted and the mRNA levels of GADD153 and caspase-12 were detected by Real-time PCR. The total proteins were used to determine the protein levels of GADD153, caspase-12, Bax, Bcl-2, pro-caspase-3 and cleaved caspase-3, representative western blot images and quantification analysis normalized to the β-actin and control, respectively, are from three independent experiments. (A) Effects of lycopene on H/R-induced GADD153 mRNA expression. (B) Effects of lycopene on H/R-induced CHOP/GADD153 protein expression. (C) Effects of lycopene on the ratio of Bax and Bcl-2 in cardiomyocytes. (D) Effects of lycopene on H/R-induced caspase-12 mRNA expression. (E) Effects of lycopene on pro-caspase-12 and cleaved caspase-12 in H/R-treated cardiomyocytes. (F) Effects of lycopene on pro-caspase-3 and cleaved caspase-3 in H/R-treated cardiomyocytes. Data represent the mean ± SEM. *P<0.05, **P<0.01 versus control; ^***#***^ *P*<0.05, ^**##**^ *P* <0.01 versus H/R group. (Lyc, Lycopene; H/R, hypoxia/ reoxygenation).

It has been demonstrated that excessive ER stress in I/R-injury would initiate the apoptotic signaling of ER stress [7, 9], we therefore investigated the expression and activity constituents of ER stress-induced apoptotic pathway. We firstly evaluated the CHOP/GADD153 mediated signaling pathway, our data showed that lycopene significantly down-regulated the expression of CHOP/GADD153 mRNA and proteins enhanced by H/R (Fig 5A and 5B), and subsequently markedly decreased the ratio of Bax to Bcl-2 protein expression (Fig 5C). Studies have shown that ER stress may induce apoptosis by caspase12-dependent apoptotic mechanisms [5]. It remains to be defined whether lycopene alleviated ER stress-induced apoptosis through inhibiting the caspase-12 signaling pathway, we consequently tested two important biochemical markers of this pathway. We observed that pre-incubation of cardiomyocytes with lycopene significantly down-regulated the expression of caspase-12 mRNA (Fig 5D) and cleaved caspase-12(Fig 5E), cleaved caspase-3 (Fig 5F) compared with H/R-treated cells.

### Lycopene alleviates TG-induced ER stress and injury in cardiomyocytes

**Fig 6:**
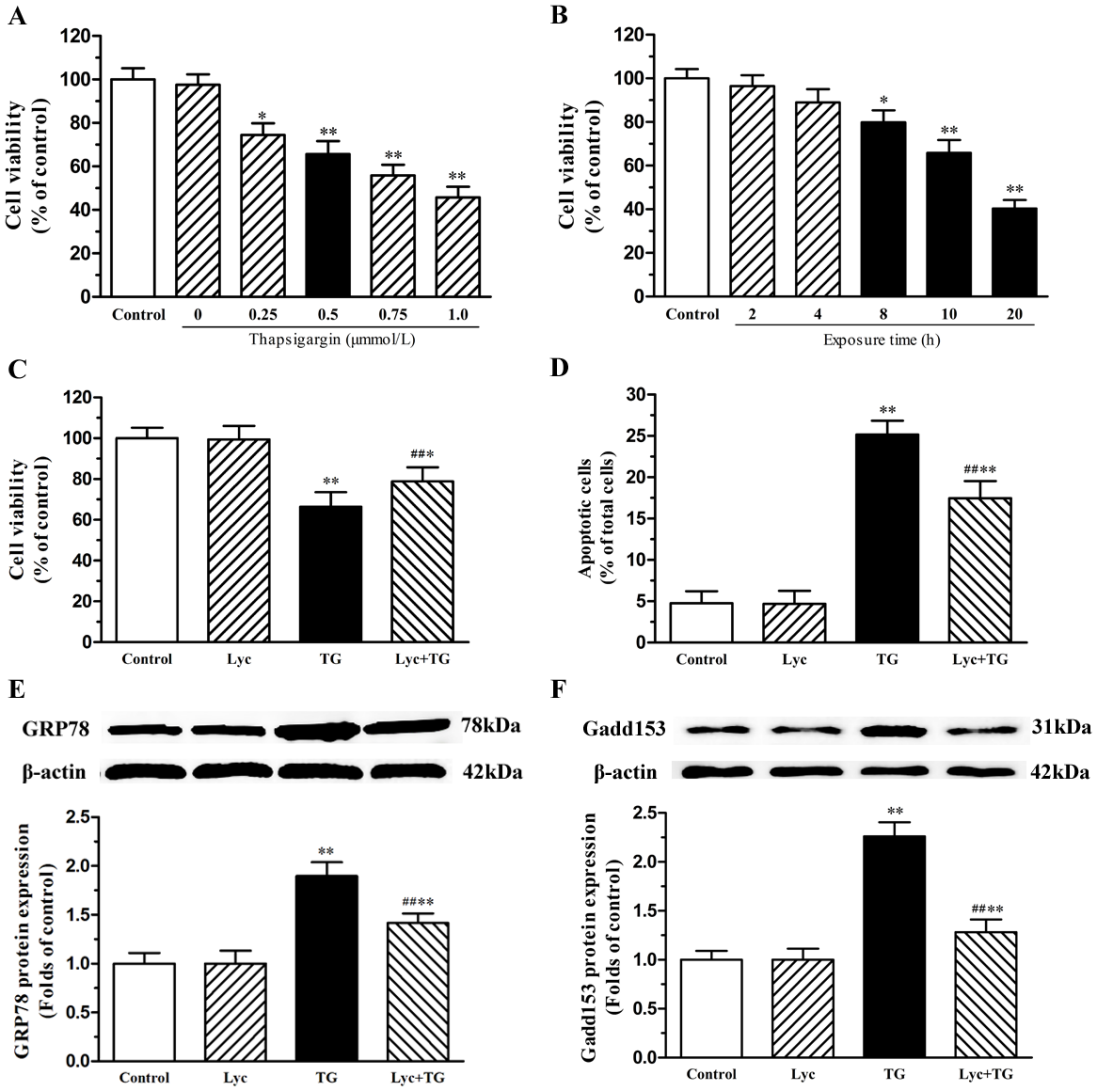
Effects of lycopene on ER stress inducer (thapsigargin, TG)-induced changes in neonatal cardiomyocytes. Cell viability was detected by CCK-8 assay. The total proteins of cardiomyocytes were extracted and the protein expression levels of Bip/GRP78 and GADD153 were detected by western blot. (A) TG evoked a dose-dependent decrease in the viability of neonatal cardiomyocytes. Cells were pretreated with TG (0.25, 0.5, 0.75 and 1.0μM) for 10h, respectively. (B) TG-mediated loss of cell viability increased with treatment duration. Cells were treatment with 0.5μM TG for 2, 4, 8, 10 and 20h, respectively. (C) Lycopene reversed TG-caused loss of cell viability. Cells were pretreated with or without 0.5μM TG for 10h after the lycopene pretreatment. (D) The relative proportion of TUNEL-positive cells is shown in each group. Representative western blot image, quantitative analysis normalized to the β-actin and control respectively showed the expression of GRP78 (E) and GADD153 (F). These assays were repeated three times. Values are mean ± SEM. *P<0.05, **P<0.01 versus control; ^**##**^ *P* <0.01 versus H/R group. (Lyc, Lycopene; H/R, hypoxia/ reoxygenation).

To further confirm whether lycopene could alleviate ER stress and ER stress-induced injury, we used thapsigargin (TG), a commonly used agent known to rapidly elicit ER stress in cardiomyocytes. Cardiomyocytes were exposed to a range of TG concentrations from 0.25 to l.0μM for 10h to find an optimal dose for subsequent assays (Fig 6A). TG evoked a dose-dependent decrease in the cell viability of cardiomyocytes, among that treatment with 0.5μM TG reduced cell viability to 65.67% of control, while l.0μM TG decreased cell viability to 45.71% of control. As illustrated in Fig 6B, 10 and 20h treatment with 0.5μM TG markedly reduced cell viability to 65.83% and 40.34% of control, respectively, whereas 2 and 4h treatment had no significant effects. According to these results, treatment with 0.5μM TG for 10h was chosen for the following experiments. As shown in Fig 6C, compared with controls treatment, 5μM lycopene pretreatment decreased the TG-induced loss of cell viability. Similarly, exposure of cardiomyocytes to TG caused an increase in the rate of apoptosis, whereas lycopene significantly reversed the changes (Fig 6C and 6D). Compared with controls, the protein expression of GRP78 and CHOP/GADD153 have been significantly elevated with 0.5μM TG pretreatment, conversely, lycopene markedly alleviated TG-induced increase in GRP78 and CHOP/GADD153 protein expression of cardiomyocytes (Fig 6E and 6F).

## Discussion

ER stress induced apoptosis plays an important role in myocardial I/R-injury [9, 37]. ROS is considered as a major factor in initiating ER stress in various cells [9, 38] and serves as a critical mediator in I/R-induced apoptosis [39], consequently, identifying naturally and nontoxic orally antioxidants that are able to attenuate myocardial I/R-injury is still a focus. Lycopene, a most effective antioxidant among the carotenoids, has been well known to display potent antioxidant and cardioprotective properties [12, 18, 40]. However, the mechanisms underlying its protective effects in I/R-injury remain unclear. The present work demonstrated that lycopene exerts cardioprotective effects against H/R-injury as revealed by improving cell viability and decreasing apoptosis, reducing ROS generation and enhancing the level of phosphorylated AMPK, ameliorating ER stress and ER stress-induced apoptosis in H/R-treated cardiomyocytes.

ROS generation is recognized as an early event in the process of myocardial I/R [9] and some studies have shown that cardiomyocytes H/R causes ROS generation [12, 41]. It has been demonstrated that ROS could be inhibited by lycopene in cerebral I/R-injury [15]. In our study, we confirmed that H/R induced ROS production and lycopene significantly inhibited H/R-induced ROS generation (Fig 3A–3D). It has been reported that ROS plays a vital role in regulating the activity of AMPK [21] and triggering ER stress [9]. Increasing evidences have demonstrated that AMPK inactivation-mediated ER stress involved in and contributed to I/R-injury, and activation of AMPK followed by inhibiting ER stress could reduce myocardial I/R or H/R injury [22–24]. Previous study showed that lycopene could efficiently prevent the H/R-induced a reduction in the cellular ATP concentration [12] which is associated with AMPK activation. A recent report showed that lycopene could regulate GRP78 expression which is a marker of ER stress in prostate cancer [14]. Here, we observed that H/R treatment decreased the phosphorylation of AMPK, increased the mRNA expression of GRP78, ATF6, sXbp-1 and eIF2α, up-regulated the GRP78 protein expression, the level of phosphorylated eIF2α and the cleaved ATF6 expression (Fig 4A–4I), whereas lycopene significantly up-regulated the protein expression of p-AMPK, inhibited H/R-induced the expression of GRP78, ATF6 mRNA, sXbp-1 and eIF2α mRNA as well as p-eIF2α in cardiomyocytes (Fig 4A–4H). These results indicate that the protective effect of lycopene against ROS-induced injury is closely connected with the level of phosphorylated AMPK and ER stress in the H/R-injury.

Previous studies showed that ER stress contributes to cardiomyocyte apoptosis after myocardial I/R through the CHOP and caspase-12 signaling pathway [23, 24]. CHOP/GADD153 sensitizes cells to ER stress induced-apoptosis via causing the imbalance of Bcl-2 family members and then regulating cytochrome *c* release from mitochondrial to activate apoptotic cascade [9]. In this study, H/R treatment significantly increased CHOP/GADD153 expression as well as the ratio of Bax to Bcl-2 (Fig 5). Numerous studies have shown that lycopene may exert its protective effect via maintaining the balance of Bax and Bcl-2 in cortical neurons, prostate cancer cells, hepatic tissue and kidney tissue [14, 15, 42, 43]. Our previous study reported that lycopene strikingly inhibited cytochrome *c* release and subsequent cell apoptosis [12]. We found that in this study lycopene decreased CHOP/GADD153 expression and ameliorated the H/R-induced Bax/Bcl-2 ratio elevation in cardiomyocytes (Fig 5). These findings suggest that lycopene inhibited the apoptosis such as mitochondria-dependent apoptotic pathway probably via attenuating the ER stress and ER stress induced apoptosis.

ER stress-specific caspase-12, normally exists in an inactive pro-caspase form, plays a pivotal role in initiating ER stress-induced apoptosis via activating caspase-9 and caspase-3[44]. It has been described that caspase-12 or caspase-3 were cleaved in reperfusion of ischemic myocardium [45]. In our study, we observed that H/R significantly increased caspase-12 mRNA expression, caspase-12 and caspase-3 activity (Fig 5). Some reports showed that lycopene markedly inhibited caspase-9 or caspase-3 activity induced by relative pathological stimuli in hepatocytes and cardiomyocytes [46, 47]. We detected that lycopene inhibited H/R treatment triggering increase in the expression of caspase-12 mRNA, activation of caspase-12 and caspase-3 in primary cultured neonatal mouse cardiomyocytes (Fig 5). These results suggest that caspase-12 pathway is involved in the lycopene protection against H/R-injury and lycopene inhibited caspase-3 activity may be implicated in alleviating the activation of caspase-12.

Despite the established roles of ER stress-induced apoptosis of cardiomyocytes in H/R-injury [23, 24], there is no report on the effects of lycopene on ER stress-induced injury in cardiomyocytes. Given the defined role of ER stress and the related pathological conditions of H/R-injury, we propose that in addition to triggering ER stress via disruption of protein proper folding, TG also propagates ER stress, which is supported by both previous reports [8, 48] and our findings that TG treatment resulted in an increase in GRP78 and CHOP/GADD153 expression along with the rate of apoptosis. In our current study, we found that TG treatment could decrease the cell viability of cardiomyocytes which was similar to the H/R treatment. These results suggest that TG elicited ER stress may mimic the responsible for H/R-injury. At the same time, we found that pretreatment with 5μM lycopene significantly improved TG-induced the loss of cell viability and decreased TG-induced apoptosis in cardiomyocytes, alleviated the protein expression of GRP78 and CHOP/GADD153 in TG-treated cardiomyocytes. These results suggest that lycopene protects primary cultured neonatal mouse cardiomyocytes against H/R-injury is, at least in part, through suppressing ER stress-induced injury.

In conclusion, our present study demonstrated that the protective effects of lycopene on H/R-injury in primary cultured neonatal mouse cardiomyocytes. Our evidences suggest that the protective effects of lycopene on mitochondria-dependent apoptotic pathway might be associated with suppression of the ER stress and ER stress-induced apoptosis through activating AMPK (S1 Fig). Inhibition of ER stress-induced apoptosis by lycopene may be a novel cardioprotective mechanism that could help us to establish a therapeutic approach to prevent myocardial I/R-injury. Nevertheless, our studies were only limited to a cellular model, and further *in vivo* or clinical studies are needed to confirm the cardioprotective effects of lycopene against myocardial I/R-injury.

## Supporting Information

S1 Fig Lycopene attenuates H/R-injury in neonatal mouse cardiomyocytes by alleviating ER stress-induced apoptosis. H/R treatment induces reactive oxygen species (ROS) generation, AMPK inactivation, and accumulation of unfolded proteins in the endoplasmic reticulum (ER) causing the activation of the ER stress-mediated UPR (activation of ATF6, IRE1, and PERK signaling pathways). Consequently, the transcription factors cleaved ATF6, spliced Xbp-1 (sXbp-1) and ATF4 are translocated to the nucleus and trigger the transcription of several genes such as the pro-apoptotic CHOP that increases the Bax/Bcl-2 ratio in mitochondria leading to activate the apoptosis effector caspase-3. On the other hand, ER stress activates caspase-12 and further activates caspase-3. Lycopene may reverse the changes by decreasing ROS production, activating AMPK, alleviating ER stress and ER stress-induced apoptosis.
(TIF)
