# Supplementary figures and images for: Lycopene Protects against Hypoxia/Reoxygenation Injury by Alleviating ER Stress Induced Apoptosis in Neonatal Mouse Cardiomyocytes
Source: PLoS One. 2015 Aug 20;10(8):e0136443. doi: 10.1371/journal.pone.0136443 (PMC4546295; doi:10.1371/journal.pone.0136443)

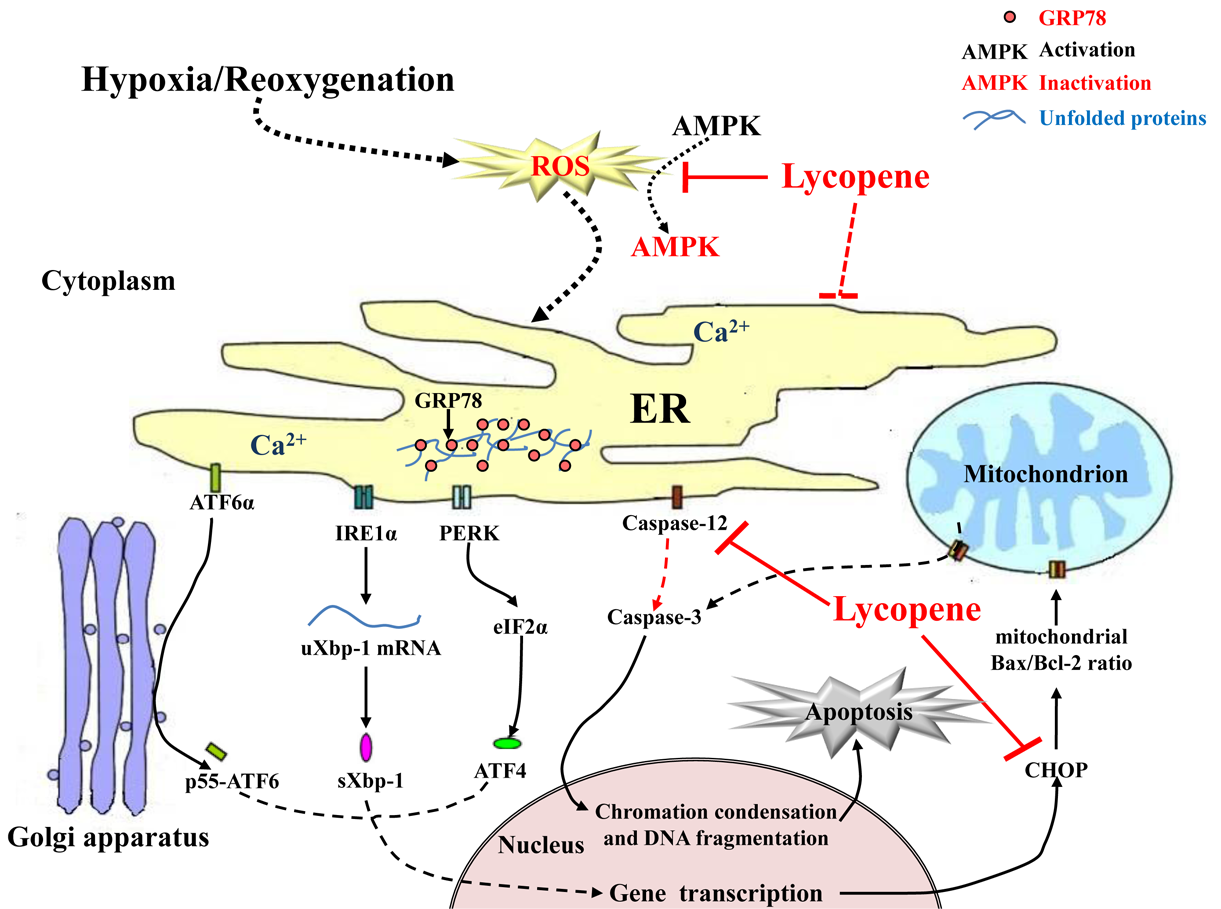

Supplement: S1 Fig — H/R treatment induces reactive oxygen species (ROS) generation, AMPK inactivation, and accumulation of unfolded proteins in the endoplasmic reticulum (ER) causing the activation of the ER stress-mediated UPR (activation of ATF6, IRE1, and PERK signaling pathways). Consequently, the transcription factors cleaved ATF6, spliced Xbp-1 (sXbp-1) and ATF4 are translocated to the nucleus and trigger the transcription of several genes such as the pro-apoptotic CHOP that increases the Bax/Bcl-2 ratio in mitochondria leading to activate the apoptosis effector caspase-3. On the other hand, ER stress activates caspase-12 and further activates caspase-3. Lycopene may reverse the changes by decreasing ROS production, activating AMPK, alleviating ER stress and ER stress-induced apoptosis. (TIF) [file pone.0136443.s001.tif]
